# Evaluation of physiological functions and production performance in laying hens in three different housing systems

**DOI:** 10.1016/j.psj.2025.105256

**Published:** 2025-05-17

**Authors:** Gaku Shoji, Ryota Tochinai, Shin-ichi Sekizawa, Masayoshi Kuwahara

**Affiliations:** The University of Tokyo Graduate School of Agricultural and Life Sciences Faculty of Agriculture, Japan

**Keywords:** Laying hen, Housing, Physiology, Performance, Welfare

## Abstract

In egg production management, housing systems have been one of the major issues. Although most laying hens are housed in conventional cage (CC) worldwide, alternative systems have been adopted such as enriched cage (EC) and aviary systems (AV) mainly due to concern of animal welfare of hens. To properly evaluate housing systems from production performance and welfare status, we investigated egg production, and physiological and autonomic nervous functions of hens by using a telemetry system and HRV analysis. Hens in AV showed lower productivity compared to CC and EC, and shell characteristics were quite similar between housing systems. The results also presented that egg laying rate (ELR) of EC was higher than that of CC. As physiological parameters, we continuously recorded an electrocardiogram (ECG), heart rate (HR), body temperature (BT) and locomotor activity (LA) of hens in three different housing systems by a telemetry system. Also, to evaluate autonomic nervous function of hens as a way to investigate stress and welfare of hens on poultry farms, we analyzed heart rate variability (HRV). Hens in all three groups showed the diurnal HR and LA patterns. CC and EC had also diurnal patterns of autonomic nervous functions, suggesting that parasympathetic nervous activity is predominant during dark phase. On the other hand, these diurnal patterns were unclear in hens raised in AV, which implies that AV may suppress parasympathetic nervous activity of hens during the dark phase. Therefore, AV might diminish the rest quality of hens, and lower the biological functions and recover of hens. In addition to that, hens in AV did not increase LA by quantitative analysis. It can be concluded that AV could be harmful to egg production and welfare of hen, and that EC system seems to be a more reasonable alternative option for conventional egg production from both egg productivity and animal welfare point of views.

## Introduction

In egg production, although most laying hens are housed in CC, alternative systems have been adopted such as EC, AV and floor pens mainly due to concern of animal welfare of hens. Since installing facilities and new management methods increases production costs (He et al., 2022; Kato et al., 2022; Sumner et al., 2011), housing system has been intensively debated mainly from production performance, economics and welfare status.

Production performance of laying hen is closely related to its biological functions because of its characteristics that a laying hen produces an egg almost every day (Bain et al., 2016; Gautron et al., 2021). Thus, we evaluated production performance of laying hens in three different housing systems, CC, EC and AV.

Although behavioral studies have been often applied to evaluate welfare and stress status of laying hens (Lay Jr et al., 2011; Shimmura, et al., 2006; Shimmura et al., 2007; Tanaka and Hurnik, 1991, 1992), more studies on physiological status of hens are necessary to accurately assess welfare status. As physiological parameters, neurochemical, endocrine and immune indicators have been measured, such as plasma concentrations of serotonin and corticosterone (Pohle and Cheng, 2009), or interleukin 6 (Casalino et al., 2022) and the ratio of heterophils and lymphocytes (H/L) as immune parameters. One of the limitations in most previous studies is that invasive methods have been adopted to measure these indicators. Only physical restraint even induces high stress reactions of hens (Kjaer and Jørgensen, 2011). Another challenge is that immune parameters are affected by various physiological and behavioral conditions, and not sensitive enough to detect stress reactions of laying hens (Lentfer et al., 2015). Therefore, a tool for collecting continuous physiological data in unrestrained animals by a noninvasive method has been developed (Ahmmed et al., 2023).

The telemetric monitoring system is known as a tool for collecting physiological parameters in conscious, unrestrained animals (Cain and Wilson, 1971; Kadono and Besch, 1978). This system is more suitable than other portable equipment especially for hens in a flock because of less damage by interaction. In this study, we continuously recorded an ECG, HR, BT and LA of hens in three different housing systems, CC, EC and AV, by a telemetry system.

There is a great deal of interest worldwide in the stress and welfare of livestock, and various efforts have been made to evaluate them (Fraser et al., 2013; Hemsworth et al., 2015). We have established HRV analysis as a suitable method for investigating autonomic nervous functions by using ECG in many species (Kuwahara et al., 1999, 1994, 1996; Ishi et al., 1996; Akita et al., 2002, 2008; Yoshida et al., 2015). In addition to it, HRV analysis is considered to be a useful method for evaluating stress and welfare in livestock (as reviewed by Gaidica and Dantzer, 2020; as reviewed by von Borell et al., 2007), and that in poultry has recently been investigated intensively (Ahmmed et al., 2023). Therefore, here we also analyzed HRV of hens to investigate autonomic nervous activities and to evaluate stress and welfare of hens.

In summary, to properly evaluate stress and welfare of hens in three types of housing systems, CC and two alternative systems (EC, AV), from both egg productivity and physiological aspects, we investigated egg production, physiological and autonomic nervous functions of hens by using a telemetry system and HRV analysis.

## Materials and methods

### Animals and housing system

Lohmann Selected Leghorn Lite laying hens were used in this study. In total, 261 pullets were introduced to the experimental farm of the University of Tokyo at Kasama, Ibaraki, Japan at 15 weeks (103 days) of age. At first, all the pullets were reared using CC (Univent 639, Big Dutchman, Vechta, Germany) until 29 weeks (199 days) of age.

On the 29 weeks (200 days) of age, some of the hens were randomly selected and transferred to EC (Eurovent 2240-EU, Big Dutchman, Vechta, Germany) or AV (Nova 270, Big Dutchman, Vechta, Germany) (CC; *n* = 88, EC; *n* = 72, AV; *n* = 93), then those housed in each housing system until 99 weeks (687 days) of age.

Each CC contained 11 hens, providing 354 cm^2^ of floor space per hen, with 639 mm depth and 610 mm width. EC provided 1125 cm^2^ of floor space per hen, with 3618 mm depth and 1120 mm width. In the AV, the hens had free access to a 3 dimensions-space and floor, and the available area was 3017 cm^2^ per hen, with 4440 mm depth and 6320 mm width of the floor space. Hens were basically housed under the same management conditions, and a light-dark cycle (Supplementary Table 1). Feeding was conducted from 9:00 am, and water was supplied *ad libitum*. Eggs were collected daily from 1:00 pm to 2:30 pm. All the cages were cleaned up twice a day, from 10:30 am to 11:30 am, and from 2:30 pm to 3:30 pm. Feed was purchased from Showa Sangyo Co., Ltd. (Tokyo, Japan). The animal care and the experiments were performed in accordance with the Guidelines for Animal Experiments of The University of Tokyo. Protocols were approved by the Animal Research Committee of The University of Tokyo.

### Egg production and quality

Number of eggs, dead hens, daily feed (kg) and egg production (kg) were recorded daily from the beginning of group division, 29 weeks (200 days) of age, to 99 weeks (686 days) of age. ELR (%), egg weight (g), mortality (%) and feed conversion ratio (FCR) were calculated based on the totals for this period. Ten eggs from each system were collected twice at the ages of 38 weeks (265 days) and 99 weeks (687 days) to measure eggshell thickness (mm) and eggshell deformation (kg/cm^2^).

### Implanting the transmitter and data recording

A telemetric transmitter for ECG, LA and BT (easyTEL+*L*-ETA, emka TECHNOLOGIES, Paris, France) was implanted to randomly selected 18 hens on the 25 weeks (171 days) of age under isoflurane anesthesia after intramuscular administration of dexmedetomidine (0.66 μg/kg) and carprofen (5.0 mg/kg). The paired wire electrodes in a precordial bipolar lead were placed at the cervical subcutaneous region over the trapezius and the skin was closed with suturing. Bluetooth receiver mounted on the top of the room wall with Ethernet connection to computer for acquisition of signals. The ECG signals were continuously recorded by a data acquisition software (IOX2, emka TECHNOLOGIES, Paris, France). The signals of HR (bpm), BT (°C) and LA (g/s) were recorded every 5 minutes by this system. LA was calculated as the average value of the resultant acceleration of the 3-axis acceleration detected in the xyz axis over time.

### Data analysis

Recorded ECG signals were analyzed to detect RR intervals (RRI) by an ecgAUTO (emka TECHNOLOGIES, Paris, France). Power spectral analysis of HRV was performed using these RRI sets by ECG processor analyzing system (SRV-2 W, Softron, Japan), as described previously (Kuwahara et al., 1994). Briefly, RRI data were interpolated at 1 millisecond sampling interval to form a RR tachogram, in which 512 points long (Pagani et al., 1986) datasets were resampled at 100 millisecond intervals. Each set of data was applied to the Hamming window to minimize spectral leakage. A fast Fourier transform was used to obtain the power spectrum of the fluctuation. The HF component of the power spectrum is thought to reflect on the respiratory frequency, and the LF component corresponded to fluctuations of vasomotor and baroreceptor control (Akselrod et al., 1981). At rest, chicken's respiratory rate is 12 to 37 times per minute (0.2-0.61 Hz), but it increases to around 100 times per minute (1.6 Hz) in a hot environment. On the basis of these data, we defined two frequency bands of interest: LF (0.06-0.16 Hz) and HF (0.16-1.6 Hz). These bands were quite similar to the settings determined with data and respiratory sinus arrhythmia component in quail and chicks: LF (0.04-0.15 Hz) and HF (0.15-1.5 Hz) (Valance et al., 2007), which was also adapted by Kjaer and Jørgensen (2011).

### Statistical analysis

Results were expressed as mean ± standard error of the mean (SEM) or as percentages for each category. Analyses were conducted using R Statistical Software (v4.3.1; R Core Team, 2023, PBC, Boston, USA). Fisher's exact test with adjustment of P values by Benjamini–Hochberg method was used to ELR. Survival rate (mortality) was evaluated by log-rank test with adjustment of P values by Bonferroni method. Other data were analyzed by one-way ANOVA for CC, EC and AV groups. As the F value for effect of housing system was significant, Turkey-Kramer was further applied for evaluation of differences among the means. Students’ paired t-test was employed for comparing means of paired variables. A value of *P* < 0.05 was considered significant.

## Results and discussion

### Egg production and quality

ELR, egg weight, mortality, and FCR were calculated (Table 1). EC showed the highest ELR (*P* < 0.05), and ELR in CC was higher than that in AV (*P* < 0.05). Egg weight values were almost same in three groups (CC; 63.53 g, EC; 62.52 g, AV; 63.65 g). Although there were no significant differences between the three groups in mortality, mortality of CC was the highest and that of EC was the lowest (CC; 17.0 %, EC; 6.9 %, AV; 8.6 %). FCR of EC was the lowest and that of AV was the highest (CC; 1.985, EC; 1.922, AV; 2.260).

**Table 1:** Production performance of hens housed in three different housing systems from 29 weeks (201 days) to 99 weeks (686 days) of age.

	CC	EC	AV
Egg laying rate (ELR) (%)	81.0 % ^a^	86.0 % ^b^	75.3 % ^c^
Egg weight (g)	63.53	62.52	63.65
Mortality (%)	17.0 %	6.9 %	8.6 %
Feed conversion ratio (FCR)	1.985	1.922	2.260

^abc^Different letters indicate significant differences between the housing systems (*P* < 0.05).

Eggshell thickness from AV was higher than that from CC at 38 weeks (265 days) of age (*P* < 0.05) (Table 2). On the other hand, eggshell deformation from AV was lower than those from CC and EC at 99 weeks (687 days) of age (*P* < 0.05).

**Table 2:** Egg quality of hens in three different housing systems on 38 weeks (265 days) and 99 weeks (687 days) of age.

age (weeks)	Shell thickness (mm)			Shell deformation (kg/cm^2^)		
	CC	EC	AV	CC	EC	AV
38	0.381 ± 0.01 ^a^	0.388 ± 0.01 ^ab^	0.409 ± 0.01 ^b^	4.94 ± 0.20	5.29 ± 0.19	4.87 ± 0.32
99	0.356 ± 0.01	0.352 ± 0.02	0.344 ± 0.01	4.37 ± 0.15 ^a^	4.61 ± 0.24 ^a^	3.59 ± 0.22 ^b^

^ab^Different letters indicate significant differences between the housing systems (*P* < 0.05).

Egg production and quality of egg in each housing system are affected by factors like environmental conditions, management and handling (El-Sabrout et al., 2022). In this study, hens in AV showed lower productivity compared to CC and EC, and shell characteristics were quite similar between housing systems, which seems to agree with most previous studies (summarized by Philippe et al., 2020). In addition, mortality of current study can be considered as typical in Japan based on the previous survey (Kato et al., 2022). Hence, the present performances of hens in three housing groups appear to coincide with most previous studies, which indicates that each housing system and management of this study can be regarded as general conditions. On the other hand, since EC showed lower mortality, which also contributed to lower FCR compared to CC, egg production of EC as one of the alternative systems was higher than that of CC based on the results of ELR and mortality.

### Changes of physiological and HRV parameters of hens in three housing systems

The present study mainly aimed to compare physiological functions of hens raised in three different housing systems, CC, EC or AV. Over the past decades, physiological data such as HR, BT, LA and blood pressure of broiler chickens or laying hens have been obtained by using a telemetry system (Cain and Abbott, 1970; Cain and Wilson, 1971; Kadono and Besch, 1978; Savory et al., 2006). In these previous studies, however, each bird was housed individually in a cage. Since hens are reared in a group even in CC under commercial conditions, in the current study, we recorded an ECG, HR, BT and LA of free-moving hens housed in a group. Besides, this is the first attempt to compare these physiological functions of hens in three different housing systems. Here, we applied a telemetry system, which could be suitable for hens in group-housing compared with other portable equipment, like holter systems, considering damage by interaction.

We have applied HRV as a suitable method for investigating autonomic nervous functions to many animal species (Kuwahara et al., 2004; Yamamoto et al., 2009; Yoshida et al., 2015). The main advantage is that it can provide a convenient index of parasympathetic and sympathetic interactions, both continuously and non-invasively from unrestrained animals, including farm animals (Kuwahara et al., 2004; Yoshida et al., 2015, 2023; Bun et al., 2018; Aoki et al., 2020). Furthermore, HRV analysis is applied to evaluate stress and welfare in livestock (as reviewed by Gaidica and Dantzer, 2020; as reviewed by von Borell et al., 2007). Based on these previous studies, we interpreted HF as a useful indicator of parasympathetic nervous system function, and LF/HF as the balance index between sympathetic and parasympathetic nervous activity.

However, very few previous studies analyzed HRV in poultry, and most of the studies recorded ECG for a short period to assess acute stress (Kjaer and Jørgensen, 2011; Korte et al., 1998). One of the main reasons is that it is difficult to obtain high-quality data in birds, which can be analyzed for HRV, especially by non-invasive equipment (as reviewed by von Borell et al., 2007). Even though, since further research has been required to assess more accurate welfare status of hens, HRV in poultry has been intensively targeted for investigation (Ahmmed et al., 2023). Then, here we analyzed HRV of hens housed in groups in three different housing systems to investigate autonomic nervous functions and welfare status.

**Fig. 1:**
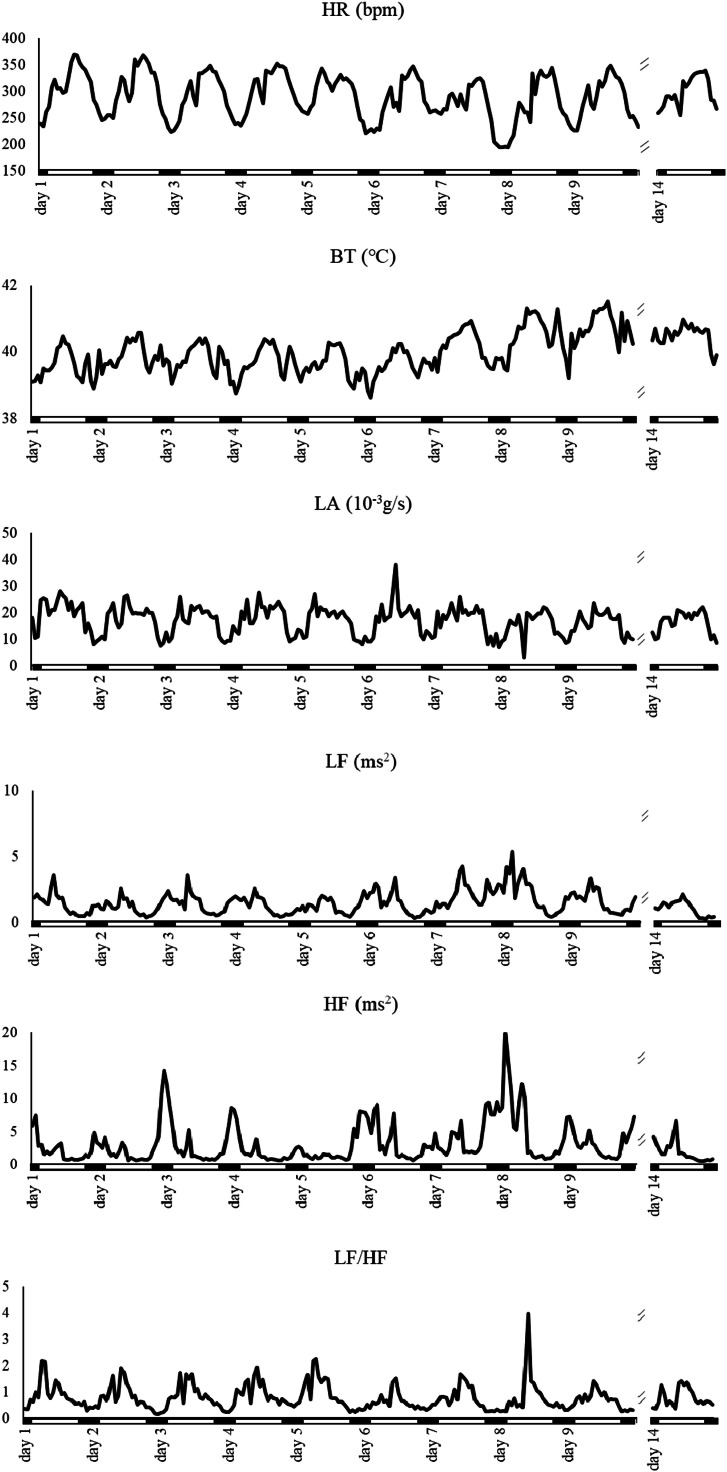
Representative changes of physiological function and HRV indexes in hens in CC from 1 day to 9 days and 14 days after group division. Lines are the means of hourly data.

Although some behavioral studies have investigated the period for hens to adjust to the housing environment (Shinmura et al., 2006; Tanaka and Hurnik, 1991), no studies have ever tried to examine this problem from the physiological point of view. In this study, therefore, we evaluated the period for hens to adjust to the housing environment by parameters of physiological functions and autonomic nervous functions of hens, by using a telemetry system and HRV analysis. We observed the changes of HR, BT, LA and HRV parameters (LF, HF, LF/HF) of hens in 1∼9 and 14 days after group division. Representative changes of hens in CC are presented in Fig. 1. HR, BT and LA values clearly showed diurnal rhythms, the values in the light phase were higher than those in the dark phase from 1 day after the division. HR in the dark phase decreased gradually with time after group division. As for HRV values, HF in the dark phase was highly maintained, and LF/HF value presented diurnal rhythms during this period which in the light phase was higher than that in the dark phase. Although temporary increases and decreases were observed, the patterns of these values did not present marked changes with time after the division.

**Fig. 2:**
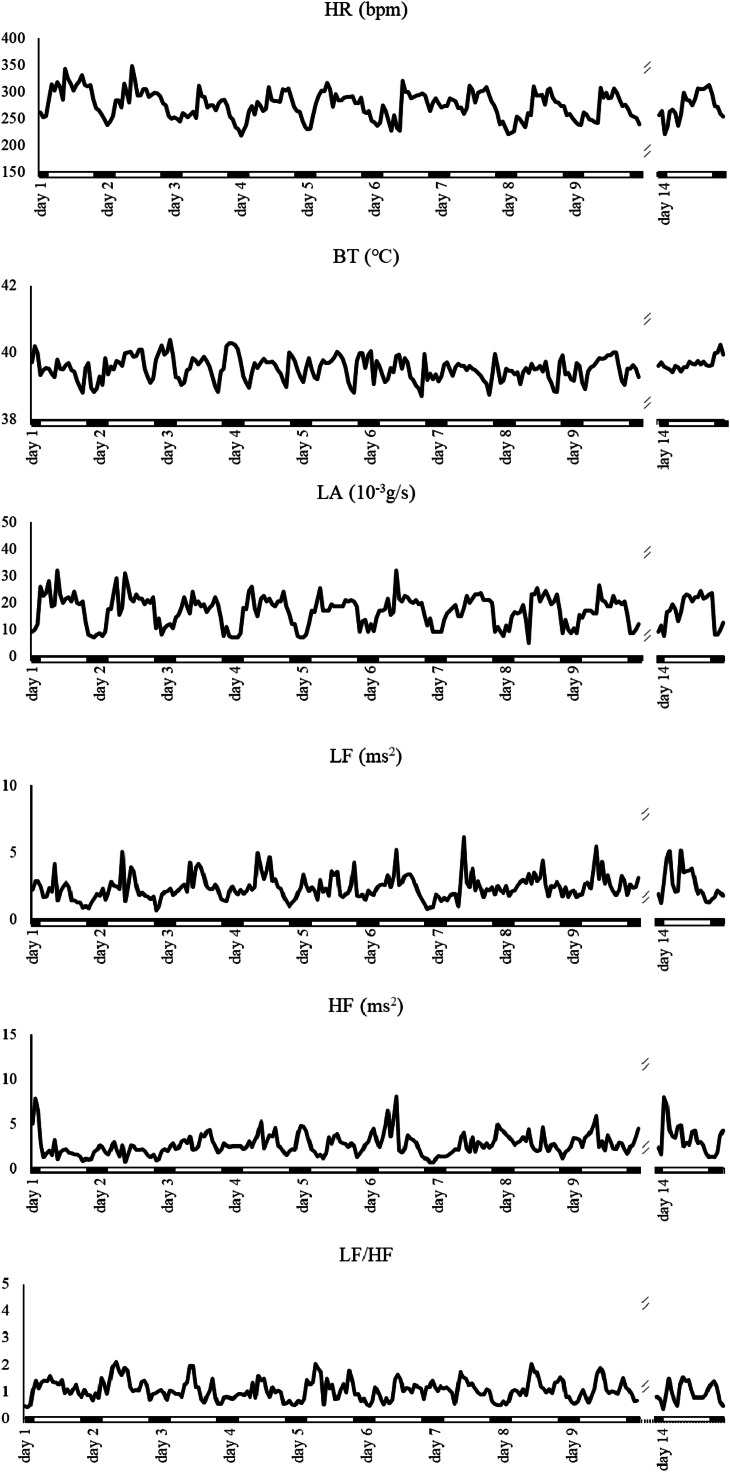
Representative changes of physiological function and HRV indexes in hens in EC from 1 day to 9 days and 14 days after group division. Lines are the means of hourly data.

Representative changes of hens in EC are shown in Fig. 2, which were almost the same as those in CC, and the change patterns of parameters were basically constant during this period. The decrease in HR in the dark phase was smaller in EC compared to that in CC.

**Fig. 3:**
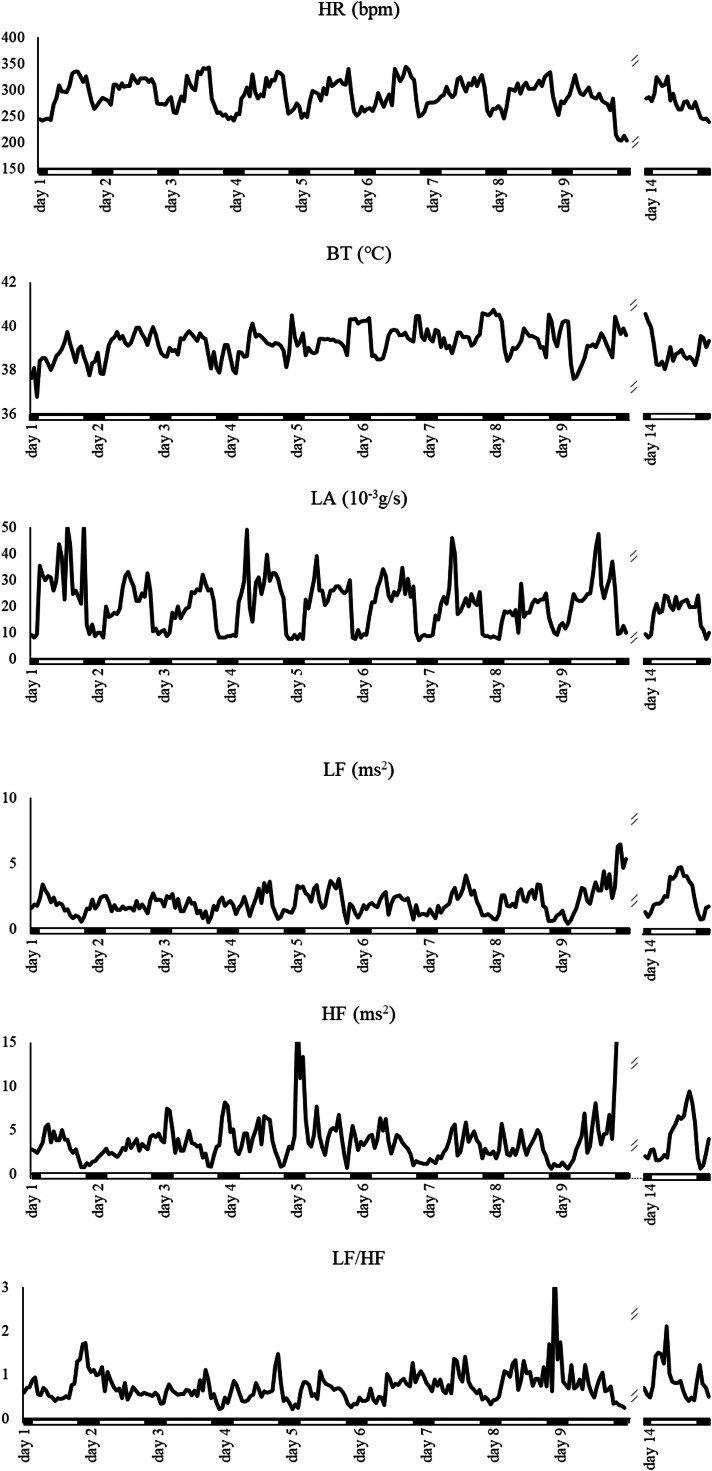
Representative changes of physiological function and HRV indexes in hens in AV from 1 day to 9 days and 14 days after group division. Lines are the means of hourly data.

Fig. 3 displays representative changes of hens in AV. Even though HR and LA values showed diurnal patterns, HR in the dark phase did not decrease over time. Sudden increases of LA were frequently observed in the light phase, especially 1 day after the group division. After 5 days of division, BT in the light phase increased, and the diurnal pattern of BT became unclear. Also, HRV values of hens in AV presented different patterns from those in CC and EC from around 5 days after the division. HF in the dark phase gradually decreased, and even became lower than that in the light phase. LF/HF showed a smaller decline in the dark phase with time, and LF/HF in the dark phase was finally higher than that in the light phase. Although there were temporary increases and decreases in these parameters, the diurnal patterns were basically constant by 1 week after the group division (Figs. 1, Fig. 2, Fig. 3). It indicates that after the introduction to each housing system, hens which were reared in CC could gradually adjust to the new environment such as EC and AV, and exhibit distinct physiological traits in each system within a week. Previous behavioral studies indicated that it was not constant for hens to adjust to the new environment, from a few days to 2 weeks, which seems to be affected by various factors, such as age of hens, prerearing conditions and behavioral parameters used for analyses. Thus, it must be noted that the period required for adjustment in this study was under the conditions of this experiment. Shimmura et al. (2006) showed that it took longer for hens to adjust to AV compared to EC because of the environmental complexity. In this study, although hens in AV presented temporary increases of LA on 1 day after the group division, and AV seems to have a greater influence on BT and HRV parameters compared to CC and EC, it took almost same period for hens to present distinct physiological traits in AV compared with hens in CC and EC.

**Fig. 4:**
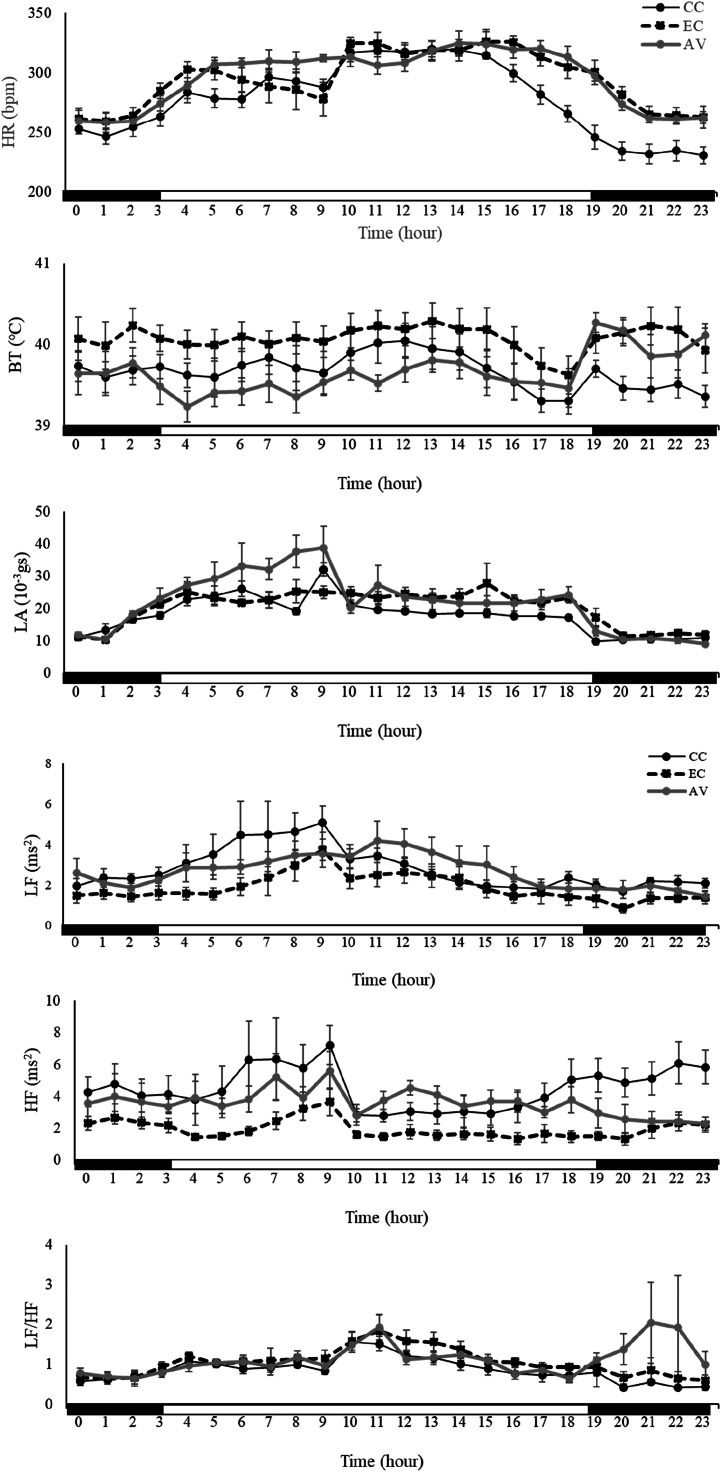
Comparison of hourly values of physiological function and HRV indexes in hens in three different housing systems. Circles and lines are mean ± SEM.

Hence, in the present study, we compared the physiological and HRV parameters of hens in different housing systems on 8 days after the group division, which are shown in Fig. 4. In addition to it, Table 3 displays the values of three groups in both the light and dark phases.

**Table 3:** Light- and dark-phase physiological function and HRV in hens in three different housing systems.

Measure	CC		EC		AV	
	Light	Dark	Light	Dark	Light	Dark
HR (bpm)	296.5 ± 4.9	231.0 ± 6.4 ^* A^	300.4 ± 9.2	264.8 ± 8.1 ^* B^	311.9 ± 5.5	264.0 ± 3.2 ^* B^
BT (°C)	39.7 ± 0.27	39.5 ± 0.11	39.9 ± 0.18	40.0 ± 0.22	39.4 ± 0.17	40.0 ± 0.13
LA (10^-3^gs)	18.2 ± 0.46 ^a^	10.8 ± 0.44 ^* A^	22.0 ± 1.27 ^ab^	13.5 ± 0.85 ^* B^	23.2 ± 1.81 ^b^	10.9 ± 0.58 ^* A^
LF (ms^2^)	2.77 ± 0.34	2.29 ± 0.21 ^A^	2.03 ± 0.37	1.39 ± 0.25 ^* B^	3.03 ± 0.63	1.83 ± 0.24 ^AB^
HF (ms^2^)	3.95 ± 0.82	6.11 ± 1.30	2.29 ± 0.50	2.86 ± 0.80	3.63 ± 0.55	3.06 ± 0.68
LF/HF	1.02 ± 0.09	0.53 ± 0.10 *	1.14 ± 0.12	0.68 ± 0.14 *	1.21 ± 0.10	1.44 ± 0.50

^ab,AB^ Different letters indicate significant differences between the housing systems (*P* < 0.05).

* Significantly (*P* < 0.05) different from light values.

HR and LA values of all three groups increased from around 3:00 am, reached the peaks around 9:00 ∼ 10:00 am, just after feeding, then gradually decreased toward 7:30 pm, the start of the dark phase. HR in CC dropped sharply after 4:00 pm, and the dark phase value was lower than those of EC and AV (*P* < 0.05). Although LA in AV was higher than that in CC, there was no significant difference compared with EC during the light phase. In the dark phase, LA in EC was higher than those in other two groups (*P* < 0.05). Between the values of the light phase and dark phase in each group, HR and LA during the light phase were higher than those during the dark phase in all groups (*P* < 0.05). BT value of CC gradually decreased to the beginning of the dark phase, which showed the diurnal pattern. EC did not display the change of BT except for the drop around the end of the light phase. Hens in AV presented increases of BT for several hours after the beginning of the dark phase. There were no significant differences between the values of the light phase and dark phase in BT in CC or EC. On the other hand, BT during the dark phase tended to be higher than that during the light phase in AV (*P* = 0.057).

As for HRV parameters, the light phase value of LF was higher than that in all three groups. The dark phase value of EC was significantly lower than that of CC (*P* < 0.05), and the light phase value of EC itself (*P* < 0.05). HF and LF/HF parameters of CC and EC presented similar variations. HF values were higher in the dark phase than those in the light phase, decreased from around 9:00 am at feeding. On the other hand, hens in AV presented increases of LF/HF, and drop of HF for several hours after the beginning of the dark phase, which made the diurnal patterns of these values unclear. Regarding the results of HRV, although the differences were not statistically significant, HF in CC and EC during the dark phase was higher than that during the light phase, and the dark phase value of CC was higher than the others. By contrast, HF in AV during the dark phase was lower than that during the light phase. LF/HF in CC and EC during the light phase were higher than those during the dark phase (*P* < 0.05). On the other hand, there was not a significant difference between LF/HF in the phases in AV, and that in the dark phase was even higher than that in the light phase.

Hens in all three groups also showed the diurnal HR and LA patterns (Fig. 4 and Table 3), as previous studies described (Cain and Abbott, 1970; Cain and Wilson, 1971; De Jong et al., 2002; Matsui et al., 2004; Savory et al., 2006). Previous studies also demonstrated the diurnal BT rhythms (Cain and Wilson, 1971; De Jong et al., 2002; Kadono and Besch, 1978; Matsui et al., 2004; Savory et al., 2006). Although CC presented the diurnal pattern of BT, BT of hens in AV during the light phase was even lower than that during the dark phase, which indicates that AV might affect the physiological functions of hens.

Non-cage system, such as AV, is regarded as desirable system in animal welfare aspects because of more space for hens that allows free activities (Ahammed et al., 2014). In this study, we quantitatively recorded LA by telemetric system. Although the resultant acceleration may not be regarded as a way of absolute determination of LA, it can be regarded as a way of relative determination. The results suggested that hens in AV may not increase movement much more than hens in cage system by quantitative analysis.

Based on the results of HF and LF/HF on 8 days after the group division, CC and EC had also diurnal patterns of autonomic nervous functions, which suggest that parasympathetic nervous activity is predominant during dark phase. It also corresponded to the lower HR and BT values with high HF value of CC during the dark phase. On the other hand, these diurnal patterns were unclear in hens raised in AV, which implies that AV may suppress parasympathetic nervous activity of hens during the dark phase (Fig. 4 and Table 3). It indicated that AV may be a stressor for hens, and that rest quality of hens in AV may be lowered during dark phase, which could be related to higher BT. Especially for several hours after the beginning of dark phase, rest quality of hens in AV might be diminished based on elevated BT and LF/HF, and decreased HF (Fig. 4). It may be estimated that sleep states affect physiological performance and recovery (Gaidica and Dantzer, 2020). In this study, the productivity of AV was lower than those of CC and EC. Although some previous studies suggested that FCR in AV may be elevated because of higher activity, hens in AV may not increase LA by quantitative analysis. Therefore, AV might lower the biological functions and recover of hens, which could be harmful to egg production, and welfare of hens on this point.

### Conclusions

This study was conducted to clarify the effects of housing systems on the physiological functions and productivity of laying hens. It is indicated that housing system may affect physiological functions, circadian autonomic nervous system changes and quality of rest in hens, which may be related to the productivity. CC and EC provided hens with parasympathetic dominance and higher quality of rest during dark phase. On the other hand, for several hours after the beginning of dark phase, parasympathetic nervous activity seemed to be suppressed and BT was increased in AV, which suggested the lower quality of rest of hens. Besides, although AV has been adopted to increase the potential for good welfare of hens because of freedom of movement, hens in AV did not increase movement much more than hens in cage systems based on our assessment of LA as a quantitative index. In summary, AV system reduces egg production, and may lower quality of the rest and welfare status. On the other hand, EC showed lower mortality, which contributes to lower FCR compared to CC. EC showed higher productivity compared to CC, and may not lower the biological functions. Therefore, it can be concluded that EC system seems to be a more reasonable alternative option for conventional egg production from both egg productivity and animal welfare point of views.

However, we cannot completely rule out the possibility that these results might be influenced by movement to a different housing system. Also, a longer period of analysis after group division should be required to evaluate long term effects of different housing systems. From these points of views, further physiological studies should be performed to assess stress and welfare of hens in different housing systems.

## Declaration of competing interest

The authors declare no conflicts of interest.

## Appendix Supplementary materials

Image, application 1
