# Supplementary material for: Evaluation of physiological functions and production performance in laying hens in three different housing systems
Source: Poult Sci. 2025 May 17;104(8):105256. doi: 10.1016/j.psj.2025.105256 (PMC12164176; doi:10.1016/j.psj.2025.105256)
Supplement: Supplementary file 1 [file mmc1.docx]

Supplementary Table 1. Changes of light period.

| age (day) | light period (hours) | light period (time) | remarks |
| --- | --- | --- | --- |
| 103 | 11.0 h | 7:00-18:00 | pullets introduction |
| 121 |  | 7:00-18:00 |  |
| 122 | 11.5 h | 6:30-18:00 | change of photoperiod |
| 129 |  | 6:30-18:00 |  |
| 130 | 12.0 h | 6:00-18:00 | change of photoperiod |
| 136 |  | 6:00-18:00 |  |
| 137 | 12.5 h | 5:30-18:00 | change of photoperiod |
| 143 |  | 5:30-18:00 |  |
| 144 | 13.0 h | 5:00-18:00 | change of photoperiod |
| 150 |  | 5:00-18:00 |  |
| 151 | 13.5 h | 4:30-18:00 | change of photoperiod |
| 157 |  | 4:30-18:00 |  |
| 158 | 14.0 h | 4:00-18:00 | change of photoperiod |
| 164 |  | 4:00-18:00 |  |
| 165 | 14.5 h | 3:30-18:00 | change of photoperiod |
| 171 |  | 3:30-18:00 |  |
| 172 | 15.0 h | 3:00-18:00 | change of photoperiod |
| 178 |  | 3:00-18:00 |  |
| 179 | 15.5 h | 3:00-18:30 | change of photoperiod |
| 185 |  | 3:00-18:30 |  |
| 186 | 16.0 h | 3:00-19:00 | change of photoperiod |
| 193 |  | 3:00-19:30 |  |
| 194 | 16.5 h | 3:00-19:30 | change of photoperiod |
| 199 |  | 3:00-19:30 |  |
| 200 |  | 3:00-19:30 | group division |
| 201 |  | from this, 3:00-19:30 in all group | |
| 202 |  |  |  |
